# Open heart surgery or echocardiographic transthoracic or percutaneous closure in secundum atrial septal defect: a developing approach in one Chinese hospital

**DOI:** 10.1186/s13019-020-01216-w

**Published:** 2020-08-06

**Authors:** Hongwei Qi, Jiangang Zhao, Xiujie Tang, Xizheng Wang, Nan Chen, Wenqing Lv, Hong Bian, Shumin Wang, Biao Yuan

**Affiliations:** 1 Cardiovascular Center, Beijing Tongren Hospital, Capital Medical University, No. 1, Dong Jiao Min Xiang, Dongcheng District, Beijing, 100730 China; 2 Department of Cardiovascular Center, The 1st Hospital, Tsinghua University, Beijing, 100016 China

**Keywords:** Atrial septal defect, Transesophageal echocardiography, Cardiac surgical procedures, Percutaneous closure, Minimally invasive closure

## Abstract

**Background:**

To study the clinical manifestations and advantages of open-heart surgery and echocardiographic transthoracic or percutaneous closure with secundum atrial septal defect (ASD). The surgeon’s learning curve was also analyzed.

**Methods:**

In all, 115 consecutive patients with ASD from May 2013 to May 2019 were enrolled. According to the operative procedure, patients were divided into three groups: group one (open repair group) (*n* = 24), where patients underwent ASD repair (ASDR) under cardiopulmonary bypass (CPB); group two (closed surgical device closure group) (*n* = 69), where patients (six patients ≤1 y and sixteen ≤10 kg) underwent transthoracic ASD occlusion under transesophageal echocardiographic (TEE) guidance; and group three (transcatheter occlusion group) (*n* = 22), where patients underwent percutaneous ASD occlusion under echocardiography. The clinical features and results of each group were analyzed. All patients were telephonically followed-up after 3 months.

**Results:**

All the three methods treating ASD were successfully performed in our hospital. It was also a typical developing history of congenital heart disease (CHD) surgery in China. One patient in the group two was transferred to emergency surgery for occluder retrieval and CPB-ASDR. Eight patients experienced failed transthoracic or percutaneous occlusion, two of whom underwent unsuccessful percutaneous closure at another hospital. Two patients each in the groups two and three were intraoperatively converted to CPB-ASDR. Two patient in the group three was converted to transthoracic occlusion surgery. All patients were discharged without any residual shunt. The three-month follow-up also did not show any residual shunt and occluder displacement.

**Conclusion:**

In low-weight, infants, or huge ASDs with suitable rim for device occlusion, transthoracic ASD closure was successfully performed. Based on knowledge of ASD anatomy and skilled transthoracic occlusion of ASD, surgeons can perform percutaneous occlusion of ASD under echocardiographic guidance.

## Background

Since the early reports of surgical atrial septal defect (ASD) closure in 1948 (without direct visualization) [1] and in 1952 (with hypothermia and inflow occlusion) [2], followed by completion of the first ASD repair (ASDR) under cardiopulmonary bypass (CPB) in 1958. From being incurable to readily curable; reducing the impact of CPB to avoiding CPB; median sternotomy to cosmetic lateral incision [3]; large incision to small incision [4, 5]; and thoracoscopy-assisted [6] to robotic surgery [7]; radiographic percutaneous closure [8, 9] to transesophageal echocardiography (TEE)-guided transthoracic small incision ASD closure [10], TEE-guided percutaneous ASD occlusion, and transthoracic echocardiography (TTE)-guided percutaneous ASD occlusion, all while ensuring the efficacy, and reducing damage to the body, ASD treatment has been a significant aspect of the history cardiovascular surgery. We analyzed the data on ASDR, TEE-guided transthoracic ASD occlusion, and TEE combined with TTE-guided percutaneous ASD occlusion in our hospital using a retrospective and prospective analyses. In addition to being a clinical report, this study also discusses the developmental history of ASD treatment in our hospital and the growth trajectory of the surgeon.

## Materials and methods

### Research objective: in all, 115 patients (8 m - 53 y) with first diagnosis of secundum ASD and who underwent surgical treatment from may 2013 to may 2019 in our hospital were enrolled. According to the operation procedure, they were divided into three groups

Group one (open repair group) (*n* = 24) mainly comprised patients with large ASDs, ASDs’ rim unsuitable for closure or with closure failure, and multiple ASDs. Two patients (8.70%) in this group were ≤ 1 years old, and two patients (8.70%) were ≤ 10 kg.

Group two (closed surgical device closure group) (*n* = 69) comprised patients who underwent TEE-guided transthoracic small incision ASD closure. All included patients were suitable for occlusion before December 2015. Subsequent cases were mainly young patients with low body weight who were unsuitable for percutaneous ASD closure. Six patients (9.09%) in this group were ≤ 1 years old, and sixteen patients (24.24%) were ≤ 10 kg.

Group three (transcatheter occlusion group) (*n* = 22) comprised patients who underwent ultrasound-guided percutaneous ASD closure. The selected patients were suitable for ASD closure and were of appropriate age and weight.

### Research methods

A retrospective analysis was performed of ASDR under CPB and transthoracic small incision ASD closure before December 2015.From then on, all ASD patients were assigned to the corresponding group and the appropriate surgical procedure was selected for prospective analysis after TTE assessment.

#### Group one

Thoracic access was achieved by a middle sternotomy or right parasternal incision above the third and fourth ribs. Cardiac arrest was induced under CPB. Then, the ASD was repaired with autologous pericardium, which was not fixed with glutaraldehyde.

#### Group two

**Fig. 1:**
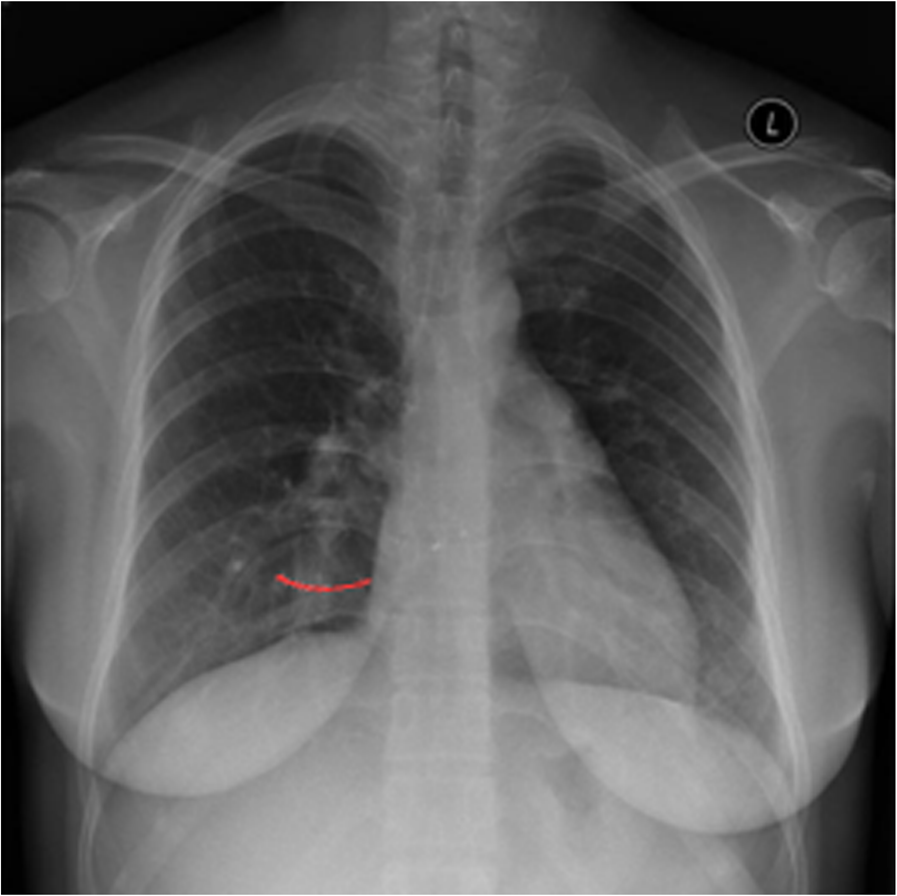
Right submammary incision

**Fig. 2:**
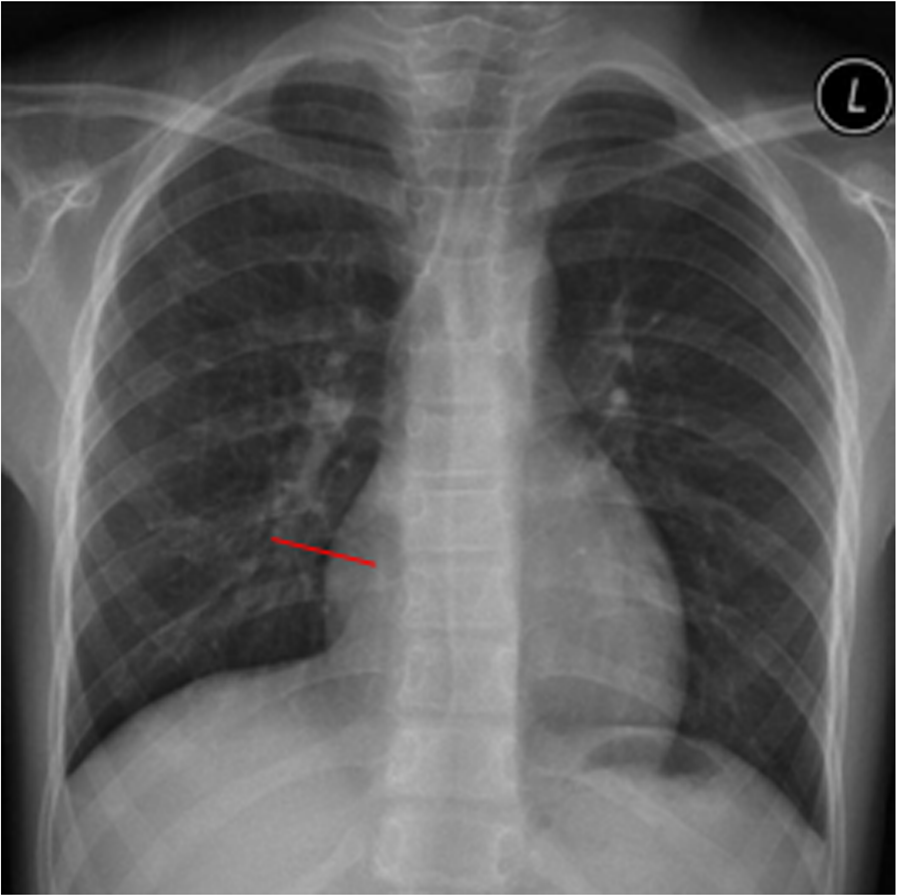
The right third intercostal space incision

**Fig. 3:**
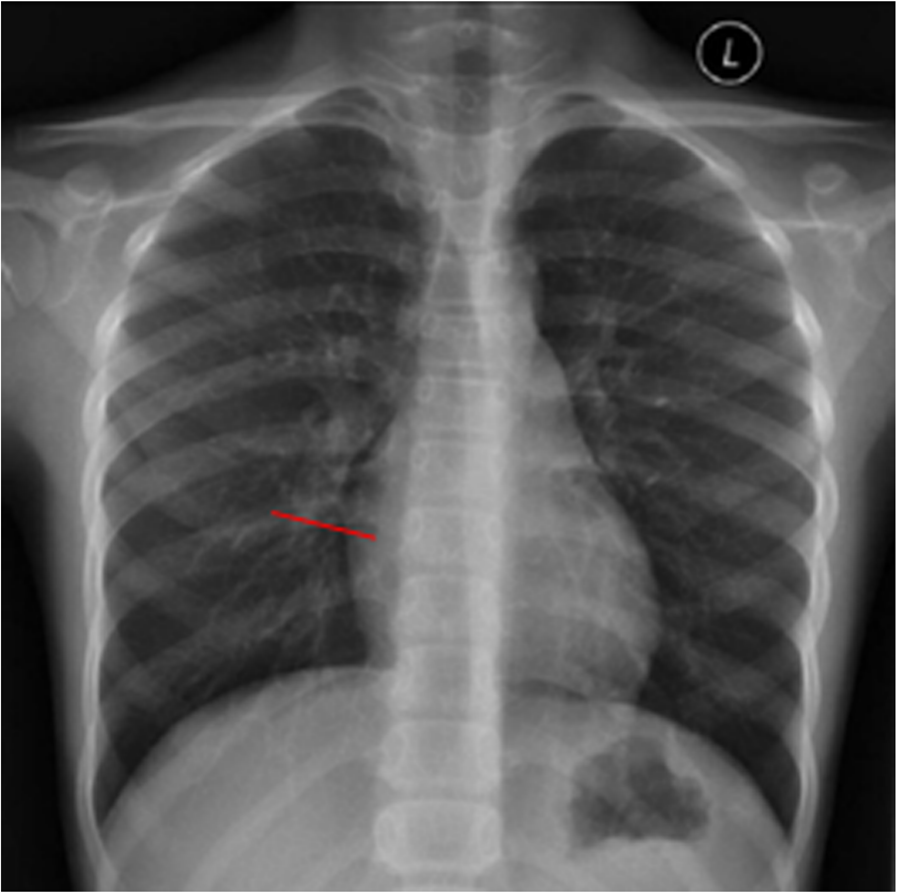
The right fourth intercostal space incision

The patient was placed in the supine position with the right chest pad placed 30° high so that the atrial septum was at a horizontal position. One mg/kg intravenous heparin was administered; no protamine was used after surgery to neutralize the effects of heparin. An ASD occluder was used (Starway Medical Technology Inc., Beijing City, China). In the middle of the umbrella surface of the right atrium, a 5–0 absorbable line was sutured for fixation. Generally, an incision was made in the right intercostal or right submammary about 2.5 to 3.5 cm (Fig. 1). The thoracic cavity was opened through third or fourth intercostal space according to the apex of the right atrium on chest radiography (Fig. 2, Fig. 3). The pericardium was opened and suspended to expose the right atrium. Two parallel 5–0 prolene purse sutures of approximately 5–10 mm in diameter were placed on the right atrium. The occluders were selected on the basis of the surgeon’s experience and in accordance with the largest diameter of the ASD. The margin of the ASD was also considered. The occluder was pushed into the sheath, and delivery sheath was inserted into the right atrium through a incision made in the middle of the purse suture. Under continuous TEE guidance, the sheath was advanced through the ASD into the left atrium. The left atrial umbrella was deployed in the left atrium first by pushing rod. Then the rod and sheath were pulled backward slightly. And then the sheath was gradually withdrawn in order to deploy the right atrial umbrella on the other side, thereby closing the ASD. A to-and-fro motion of the sheath was used to secure the device’s position across the defect. The sheath was withdrawn. The chest was closed without drainage-tube placement. After releasing the occluder, the fixing and purse lines were knotted and fixed outside the atrium. One patient was preoperatively diagnosed with pulmonary stenosis (PS); however, no PS was found during intraoperative pressure measurement. The ASDR was converted to ASD occlusion. Oral aspirin (3–5 mg/kg/d) continued for 6 months postoperatively.

#### Group three

The patient was placed in the supine position and underwent general anesthesia via a tracheal cannula. The operating racks was maintained at the level of the navel so that the doctor could perform the ultrasound examination through the chest if required. The patient was disinfected from below the navel to above the knee. The distance from the third intercostal space of the right chest to the right femoral vein puncture point was measured as a reference for “working length” of the guide wire and catheter insertion [11]. In our own opinion, the most important point was that the guide wire was founded entering right atrium through inferior vena cava in good time. Under TTE combined with TEE guidance, the long sheath was advanced through the ASD into the left atrium by rotating and push forward or pull backward slightly. The left procedure just like percutaneous ASD occlusion under X-ray. Heparin was administered intravenously for 2–3 days after surgery, and oral aspirin (3–5 mg/kg) continued for 6 months postoperatively.

### Follow-up protocol

TTE and electrocardiogram were reviewed at the local county level hospitals 3 months after discharge. All patients were followed-up by telephone.

### Statistical analysis

Statistical analysis was performed using SPSS (Version 16.0; Chicago, IL). Measurement data were expressed as mean ± standard deviation.

## Results

2.1 The age was 19.26 ± 18.40 (0.67 ~ 53.00), 9.04 ± 11.91 (0.75 ~ 53.00) and 18.40 ± 13.86 (3.08 ~ 52.00) years old in group one, group two and group three correspondingly. The weight was 36.71 ± 23.90 (5.0 ~ 70.0), 23.10 ± 17.42 (6.0 ~ 70.0) and 39.82 ± 20.02 (13.0 ~ 94.0) kg in group one, group two and group three correspondingly. In all, 115 patients were successfully treated and discharged after surgery. One patient in closed surgical device closure group suffered from occluder detachment on day 5 after operation, and emergency ASDR was performed. Eight patients experienced failed closed surgical device or transcatheter occlusion, two of whom underwent unsuccessful percutaneous closure at other hospital. Two patients each in the closed surgical device closure group and transcatheter occlusion groups were intraoperatively converted to CPB-ASDR. Two patient in the transcatheter occlusion group was converted to closed surgical device occlusion surgery. There were no surgical deaths, residual shunts, and aggravated tricuspid insufficiencies.

2.2 At the 3-month follow-up, there was no evidence of residual shunt, conduction block, or occluder displacement on cardiac ultrasound; two cases of tricuspid regurgitation were recorded.

## Discussion

ASD is a common CHD [12]. In their careers, Chinese cardiovascular surgeons start from ASD surgery and progress to more complex surgeries for CHD, valve surgery, and lastly, coronary surgery; our hospital is no exception. So far, the cardiac surgery department of the Cardiovascular Center in our hospital offers several treatment methods for ASD. We undertook closed surgical device closure and transcatheter closure under echocardiography in recent years. These different methods have their own advantages and disadvantages. Doctors who are experienced in ASDR surgery can easily understand the surgical indications of different operation methods from anatomical points. We analyzed the three operative methods of ASD in our hospital for the past 6 years. Although our data are slightly inconsistent with previous published reports, this is the actual trajectory of our doctors’ learning and the overall development of ASD treatment in our department.

Traditional treatments include open heart surgery and interventional closure. The ASDR surgical technique under CPB is very developed. The curative effect is accurate under direct vision, and the success rate is high. Almost all types of ASD can be treated in this precedure with low mortality. However, the disadvantages include long-term hospitalization, patient trauma, need for significant blood products, long-term antibiotic dependency, need for CPB, high costs, and possible adherence of the pericardium to the surface of the heart that may lead to complex future secondary surgeries. In addition, owing to the distinct and obvious scarring, patient is not willingly to accept this procedure. Eight patients in this paper experienced failed closed surgical device or transcatheter occlusion, two of whom underwent unsuccessful percutaneous closure at other hospital. These eight cases comprised huge ASDs, multiple ASDs, or unsuitable edge for occlusion.

ASD interventional occlusion has the advantage of minimal trauma and quick recovery after surgery, but X-ray cause damage to the human body. Furthermore, intraoperative use of contrast agents carries the risk of allergies and kidney failure. The TEE-guided closed surgical device occlusion circumvents the need for long incisions and CPB, but incisions are still required. Some Chinese scholars have proposed the following indications for the procedure in Chinese journals, namely when: (1) without other complicated intracardiac malformations; (2) for secundum ASDs; (3) the diameter of the septal defect is ≤40 mm; and (4) each edge of the septal defect is ≥4 mm. Several patients in this study were infant or low-weight children, and therefore unsuited for transcatheter occlusion. The closed surgical device closure also achieved satisfactory results. In addition, patients with large ASDs, especially those who had unsuccessful transcatheter occlusion, may also be suitable candidates for closed surgical device closure. We had the same successful cases in this study. Chen et al. [13] reported the reliable efficacy of the use of large occluder in mid-term follow-up. Therefore, apart from the usual indications, this procedure may be also suitable for infant, low-weight children who need treatment but are not suitable for transcatheter closure and patients with large ASDs whose margins are suitable for closure. Zhu et al. [14] reported that closed surgical device closure is more suitable for patients with ASDs who are older or have pulmonary arterial hypertension.

At present, the two more common ASD occlusion methods are slightly different with each other. The sheath travel path of transcatheter closure is much longer than that of closed surgical device closure. The angle between the delivery sheath and the atrial septum is different (transcatheter non-perpendicular vs. closed surgical device vertical), which determines whether the occluder umbrella disc and ASD are parallel or not (the transcatheter is not parallel, whereas the closed surgical device is). Therefore, it is easier to control, adjust the position, and release a closed surgical device closure, thereby improving the accuracy and safety of the operation. For large ASDs that cannot undergo transcatheter closure, closed surgical device closure may be attempted.

Mazic et al. [15] reported TEE-guided transcatheter ASD occlusion. This technique avoids the use of X-rays, but requires tracheal cannulation to avoid aspiration and affect breathing. Moreover, an esophageal probe is needed for insertion, which increases patient suffering and increases costs. Ultrasound-guided transcatheter occlusion is still in its infancy in our hospital, so the patients in this study were operated upon under general anesthesia and tracheal intubation. Both TTE and TEE were combined for transcatheter occlusion. Xu et al. [16] reported that TEE can replace X-rays with transcatheter occlusion, and the clinical effect is the same. Based on proficient TEE guidance, the development of TTE guidance is inevitable. In the case of unsatisfactory transthoracic imaging in adults, local anesthesia can be converted to general anesthesia for TEE-guided ASD occlusion. Pan et al. [11] compared TTE and TEE methods and found that TTE has the advantages of shorter operative time, shorter ventilator assist time, lower propofol usage, fewer esophageal complications, and fewer costs. In the transcatheter occlusion group, three cases were converted into other surgical methods, and they had special anatomical features: two cases of residual shunt after multiple ASD occlusion, wherein ASDR was conducted after retrieving the occluder; two case of large ASD was successfully converted to closed surgical device closure.

Many articles have reported that the successful closure of ASD in infants is safe and effective [17–19]. In this study, many patients under the age of 1 year old or weighing less than 10 kg were included in the closed surgical device closure group, and treatment effects were satisfied. Transcatheter closure studies suggest that a child’s ASD diameter/weight ratio of less than 1.2 can be safely and effectively occluded [20, 21]. Chinese experts believe that ultrasound-guided transcatheter closure of ASD diameter ≤ 20 mm is the safest and most effective [22]. Erdem et al. [23] selected transcatheter closure under general anesthesia TEE for unsatisfactory transthoracic acoustic window, aneurysmal septal defect, and/or multiple defects.

The age of patients in the open repair group was diffused, mainly because most of the patients had unsuitable anatomy for occlusion. Patients who underwent closed surgical device closure were relatively younger. Moreover, most of these patients relied on financial aid and lived in remote areas. Because waiting for the appropriate age and weight for transcatheter ASD occlusion was inconvenient, so most of them underwent closed surgical device closure, which is also a feature of this group. If solely from a medical point of view, we agree that patients with appropriate conditions should choose transcatheter closure as much as possible. If a large ASD in infancy caused repeated respiratory infections and limited growth and development, and if the ASD is too large or in a unique location unsuitable for transcatheter occlusion, closed surgical device closure can be considered. Infants and young children with appropriate structure and ASD size can also undergo transcatheter closure after careful evaluation [17, 18]. After the failure of transcatheter closure, closed surgical device occlusion can also be tried. There were two successful cases in our study.

This study has its limitations. First, we relied on our own clinical experience and advanced experience from domestic and foreign counterparts to determine the surgical plan; thus, it is not a randomized controlled study. Second, the choice of surgical methods for some patients was determined by the wishes of the patients or their parents. While there were certain attempts at transcatheter closure and a reluctance to try closure directly with CPB-ASDR, these approaches were unlikely to be randomized control groups. In this case, it is necessary to observe the clinical effect to determine which type of surgery is suitable for the chosen patients. Third, mid-term and long-term follow-up results are lacking. We aim to continue the follow-up of this group of patients from a multi-angle observation to compare whether the mid-term and long-term efficacy is different. Cardiovascular Center in our hospital has both surgeon and cardiologist, and cardiologist presently also performs percutaneous ASD closure under X-rays. This part of the patients is not included in this paper.

We believe that echocardiography is very important in decision-making. It is necessary to accurately measure the diameter of the ASD and determine the peripheral residual size of the defect, whether to have multiple atrial defects, and whether the residual edge can fix the occluder, thereby determining ASDR or occlusion. These parameters should be considered with age and body weight together to determine whether closed surgical device or transcatheter closure is suitable for the patient. Closed surgical device closure indications are extensive, with no X-ray radiation and ease of operation and mastering. Based on the proficiency of this surgical procedure, doctors can attempt ultrasound-guided transcatheter closure. In case of strict interventional ASD occlusion indications, ultrasound-guided transcatheter ASD closure is the preferred method, followed by closed surgical device closure which can replace ASDR to some extent. Despite successful experiences, it is worthwhile to discuss whether a patient is suitable for tclosed surgical device closure after failure of interventional closure under X-ray or transcatheter closure under echocardiography. Patient-specific analysis and decision-making are critical for successful outcome.

## Conclusion

TEE-guided closed surgical device ASD closure can be used to successfully treat younger, lower-weight ASD patients and patients with large ASD with margins suitable for closure. After familiarizing with ASD anatomy and gaining proficiency in closed surgical device ASD occlusion, the surgeon can choose appropriate patients to perform ultrasound-guided transcatheter ASD occlusion to avoid incision and X-ray exposure.

## Data Availability

Datasets used or analysed during the current study are available from the corresponding author on reasonable request.

## Abbreviations

ASD: Atrial septal defect
ASDR: Atrial septal defect repair
CHD: Congenital heart disease
CPB: Cardiopulmonary bypass
PS: Pulmonary stenosis
SCI: Science Citation Index
TEE: Transesophageal echocardiography
TTE: Transthoracic echocardiography
